# Effectiveness of physiotherapy on quality of life in children with asthma: Study protocol for a systematic review and meta-analysis

**DOI:** 10.1097/MD.0000000000016195

**Published:** 2019-06-28

**Authors:** Weijian Zhang, Lilong Liu, Wenhao Yang, Hanmin Liu

**Affiliations:** a Department of Pediatrics, West China Second University Hospital, Sichuan University, Chengdu, Sichuan; b Key Laboratory of Birth Defects and Related Diseases of Women and Children (Sichuan University), Ministry of Education; c Department of Gastrointestinal Surgery, People's Hospital of Deyang City, Deyang, Sichuan, China.

**Keywords:** asthma, children, physiotherapy, quality of life

## Abstract

**Background:**

Asthma is the most common chronic lung disease in childhood, leading to a great burden to the healthcare system worldwide. Despite the medication treatment, physiotherapy is now applied for asthmatic children aiming to improve their lung function and life quality. However, the effectiveness of physiotherapy on quality of life (QoL) in children with asthma is not clear. We are aiming to perform this study to provide some evidence to doctors on asthma treatment.

**Methods:**

PubMed, Embase and the Cochrane Library will be searched from their inception to 31 March 2019 for randomized controlled trials (RCTs) published in English, which investigated the effectiveness of physiotherapy on QoL in children with asthma. Besides, additional studies will be searched by scanning the reference lists of studies and the relevant systematic reviews.

Two authors will select the studies, extract the data, and assess the risk of bias independently. Data synthesis and statistical analysis will be performed in Review manager 5.3. Stata 14.0 will be used to assess the reporting bias. Quality of evidence will be evaluated based on the Grading of Recommendations Assessment, Development, and Evaluation (GRADE) system.

**Results:**

The results will provide information on the effectiveness of physiotherapy on QoL in children with asthma and further demonstrate which physiotherapy is more effective and which domain of QoL could be improved significantly.

**Conclusion:**

The findings of this study will provide the latest evidence on the effectiveness of physiotherapy on QoL in children with asthma.

**Prospero Registration Number:**

CRD42019133181.

## 1 Introduction

Asthma is the most common chronic lung disease in childhood, characterized by airway hyperreactivity and airflow obstruction.^[1,2]^ Worldwide, asthma affects an estimated 300 million people and the prevalence is still increasing, especially in children.^[3]^ In the United State, there are around 6 million children affected by asthma, leading to a great burden on healthcare system.^[4]^ Etiology of asthma is multiple and some environmental factors related to asthma have been reported, including virus infection,^[5]^ smoking exposure,^[6]^ particulate matter, and biological allergens exposure,^[7]^ etc. Besides, genes, such as GATA3, KIAA1109, and MUC5AC, were identified associated with moderate-to-severe asthma.^[8]^

The asthma care consists of 4 important components according to the current guideline, including assessment and monitoring, education, control of environmental factors and comorbid conditions, and pharmacologic treatment.^[9,10]^ Despite the care mentioned above, physiotherapy is now applied for children with asthma aiming to improve their lung function and life quality. The widely used physiotherapies for asthma are physical training, breathing exercises, and IMT.^[11]^

Nevertheless, the effectiveness of physiotherapy on QoL in children with asthma is not clear. Moreover, no systematic review and meta-analysis was conducted to compare the 3 widely used physiotherapies for asthmatic children. Therefore, we are planning to perform this study to solve these problems.

## 2 Methods

### 2.1 Registration

We have registered this study protocol at the International Prospective Register of Systematic Reviews (registration number: CRD42019133181). The *Cochrane Handbook for Systematic Reviews of Interventions*^[12]^ will be used as a guideline to solve any problem when performing the systematic review and meta-analysis. After finishing the study, We will report it according to the Preferred Reporting Items for Systematic Reviews and Meta-Analyses (PRISMA) statement.^[13]^ No ethical statement will be required due to no direct involvement of human in this study.

### 2.2 Eligibility criteria

#### 2.2.1 Types of studies

Only RCTs will be included. Those studies should be published in English before or on 31 March 2019. We will exclude the study not written in English due to language bias.

#### 2.2.2 Types of participants

The population included should be less than 18 years old without gender or ethnicity limitations. We will exclude the studies with participants aged ≥18 years old. The diagnosis of asthma should be clearly defined on all participants in the included studies.

#### 2.2.3 Types of interventions

Physiotherapy intervention for asthma includes physical training, breathing exercises, and inspiratory muscle training (IMT). We will not consider the studies with interventions concerning pharmacology, psychology, or behaviors. Physiotherapy should be conducted for ≥2 weeks.

#### 2.2.4 Types of outcome measures

QoL will be compared between the experimental groups and the control groups. In order to evaluate the life quality, Pediatric Asthma Quality of Life Questionnaire (PAQLQ) should be used in studies and the score should be reported. PAQLQ consists of 3 domains, including symptoms, activity limitations, and emotional function.

### 2.3 Search methods

PubMed, Embase, and the Cochrane Library will be searched from their inception to 31 March 2018. The search strategy will involve the following terms: child, asthma, physiotherapy, QoL, and RCTs. The preliminary search strategy in PubMed is shown in Table 1. We will use the same search strategy for search in Embase and the Cochrane Library in accordance with different specific requirements. Additional studies will also be searched by scanning the reference lists in included studies and the relevant systematic reviews.

**Table 1:**
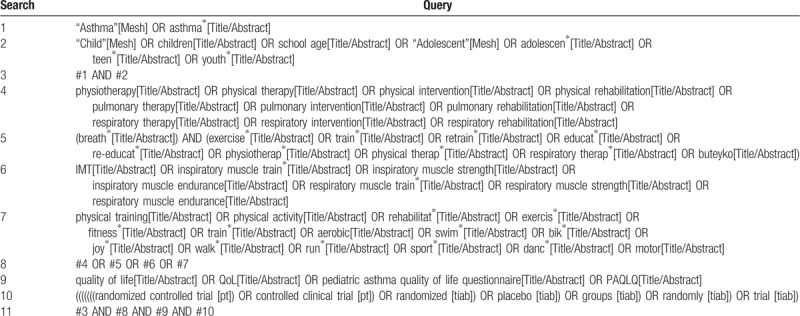
Preliminary search strategy in PubMed.

### 2.4 Study selection and data extraction

#### 2.4.1 Study selection

**Figure 1:**
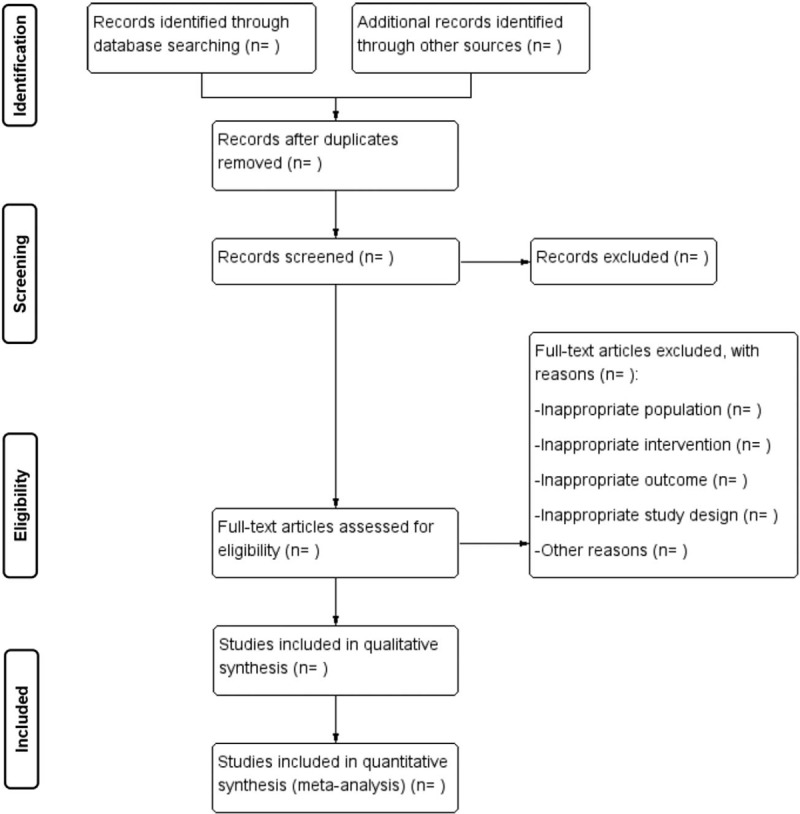
Study selection summarized in a PRISMA flow diagram.

Two authors will conduct study selection independently. The records identified through database searching and other sources will be sent to Endnote X7. After duplicates removed, records will be screened by reading the title and abstract first. If records not excluded, full-text articles assessed for eligibility will be necessary. We will include a study based on the eligibility criteria. Reasons for full-text articles excluded should be noted. Any dispute between the 2 authors will be solved by discussion or a third author. The study selection process will be summarized and reported in a PRISMA flow diagram (Fig. 1).

#### 2.4.2 Data extraction

Data will be extracted from each included study by 2 authors independently, including study characteristics (author, publication year, country, etc.), participant characteristics (number, age, severity of asthma, etc.), type and duration of intervention, score of QoL (symptoms, activity limitations and emotional function domain irrespectively). After data extracted, 2 authors will check the result with each other and any different data will be re-extracted from the study and re-checked. If different opinions between the 2 authors cannot reach an agreement, a third author will be sought for help. If data is not complete, we will contact the author. If we cannot obtain the data still, we will exclude the study depending on whether the existing data could be transformed.

### 2.5 Risk of bias assessment

The methodological quality of all included studies will be evaluated independently by 2 authors in accordance with the Cochrane Collaboration's tool.^[14]^ The following aspects will be assessed: selection bias (random sequence generation and allocation concealment), performance bias, detection bias, attrition bias, reporting bias, and other biases. The risk will be categorized into 3 levels: high level, low level, or unclear level. Any disagreement will be solved by discussion or with a third author.

### 2.6 Data synthesis and statistical analysis

#### 2.6.1 Data synthesis

Review-Manager 5.3 will be used to conduct the meta-analysis. To evaluate the effectiveness of physiotherapy on QoL, the pre- and the post- intervention score should be reported. The post-intervention score will be extracted and used for the mean difference (MD) and the 95% confidence interval (CI) calculating. The results will be weighted by using a random effect model if studies have a large diversity. We will consider it statistically significant if *P* < .05.

#### 2.6.2 Assessment of heterogeneity

We will assess the heterogeneity by the χ^2^ test and the I^2^ test. The χ^2^ test with *P* < .10 indicates statistical significance, and the I^2^ test with I^2^ > 50% indicates moderate-to-high heterogeneity.^[15]^ Reasons for the high heterogeneity will be analyzed.

#### 2.6.3 Subgroup analysis

Subgroup analyses will be conducted to investigate the effectiveness of physiotherapy on life quality based on the type of physiotherapy (physical training, breathing exercise, and IMT), and the 3 domains of QoL (symptoms, activity limitations, and emotional function).

#### 2.6.4 Sensitivity analysis

We will carry out a sensitivity analysis by excluding individual study sequentially and compare the pooled results by using a random effect model and a fixed effect model. If necessary, low-quality studies will be excluded and the meta-analysis will be repeated to test the stability of pooled results.

### 2.7 Assessment of reporting bias

If 10 or more studies are included, we will construct a funnel plot and use the Egger test in Stata 14.0 to examine the reporting bias. The results will be calcified according to the *Cochrane Handbook for Systematic Reviews of Interventions*.^[12]^ If *P* < .1, we will consider it a significant reporting bias.

### 2.8 Confidence in cumulative evidence

We will assess the quality of evidence by using the GRADE system. Quality of evidence will be adjusted to a high level, moderate level, low level, or very low level.

## 3 Discussion

Physiotherapy such as physical training has been proved to be beneficial for healthy children by improving their musculoskeletal health and mental health.^[16,17]^ However, the effects of physiotherapy in asthmatic children remain uncertain. Previous publications have reported the effects of physiotherapy in patients with asthma, but these studies included both children and adults, or adults only.^[11,18,19,20]^ Although some systematic reviews did not include adults, they only investigated the effects of one type of physiotherapy.^[21,22,23,24]^ Thus, we are going to include different types of physiotherapy and asthmatic children only to conduct a systematic review and meta-analysis. This systematic review will calcify the effectiveness of physiotherapy on QoL in asthmatic children and demonstrate which physiotherapy is more helpful. It will provide evidence directly to the doctors for making a choice on asthma treatment.

Some limitations will exist because blinding of participants involving physiotherapy is not possible, leading to a decrease in the quality of evidence in this study probably. Besides, language bias may exist since studies published in a language other than English will be excluded.

## Author contributions

WJ Zhang put forward the concept and drafted this protocol. WJ Zhang, LL Liu, and WH Yang will conduct the study search, study selection, and data extraction. WJ Zhang and LL Liu will analyze the data and assess the risk of bias. HM Liu will help to solve any dispute in this study. WJ Zhang and LL Liu are co-first authors who contributed equally to this study. All authors reviewed and approved this final manuscript.

**Conceptualization:** Weijian Zhang.

**Data curation:** Weijian Zhang, Lilong Liu, Wenhao Yang.

**Methodology:** Weijian Zhang, Lilong Liu.

**Project administration:** Hanmin Liu.

**Supervision:** Hanmin Liu.

**Writing – original draft:** Weijian Zhang.

**Writing – review & editing:** Weijian Zhang, Hanmin Liu.
